# Mobile App for Monitoring 3-Month Postoperative Functional Outcome After Hip Fracture: Usability Study

**DOI:** 10.2196/16989

**Published:** 2020-09-14

**Authors:** Merle A J Geerds, Wieke S Nijmeijer, J H Hegeman, Miriam M R Vollenbroek-Hutten

**Affiliations:** 1 Department of Trauma Surgery Ziekenhuisgroep Twente Almelo Netherlands; 2 Biomedical Signals and Systems Faculty of Electrical Engineering, Mathematics, and Computer Science University of Twente Enschede Netherlands; 3 Ziekenhuisgroep Twente Academy Ziekenhuisgroep Twente Almelo Netherlands

**Keywords:** hip fracture, remote monitoring, elderly, telemedicine, orthogeriatric, mHealth, app

## Abstract

**Background:**

As a result of an aging population, there has been an increasing incidence of hip fractures worldwide. In the Netherlands, in order to improve the quality of care for elderly patients with hip fractures, the multidisciplinary Centre for Geriatric Traumatology was established in 2008 at the Department of Trauma Surgery at Ziekenhuisgroep Twente hospital (located in Almelo and Hengelo in the Netherlands).

**Objective:**

Though the Dutch Hip Fracture audit is used to monitor the quality of care for patients with fractures of the hip, only 30.7% of patients complete registration in the 3-month follow-up period. Mobile apps offer an opportunity for improvement in this area. The aim of this study was to investigate the usability and acceptance of a mobile app for gathering indicators of quality of care in a 3-month follow-up period after postoperative treatment of hip fracture.

**Methods:**

From July 2017 to December 2017, patients who underwent surgical treatment for hip fracture were recruited. Patients and caregivers, who were collectively considered the participant cohort, were asked to download the app and answer a questionnaire. Participants were divided into two groups—those who downloaded the app and those who did not download the app. A telephone interview that was based upon the Unified Theory of Acceptance and Use of Technology was conducted with a subset of participants from each group (1:1 ratio). This study was designated as not being subject to the Dutch Medical Research Involving Human Subjects Act according to the appropriate medical research ethics committees.

**Results:**

Of the patients and caregivers who participated, 26.4% (29/110) downloaded the app, whereas 73.6% (81/110) did not. Telephone interviews with the subset of participants (n=24 per group) revealed that 54.0% (13/24) of the group of participants who did not download the app had forgotten the study. Among the group who downloaded the app, 95.8% (23/24) had the intention of completing the questionnaire, but only 4.2% (1/24) did so. The reasons for not completing the questionnaire included technical problems, cognitive disorders, or patient dependency on caregivers. Most participants in the group who downloaded the app self-reported a high level of expertise in using a smartphone (22/24, 91.7%), and sufficient facilitating conditions for using a smartphone were self-reported in both groups (downloaded the app: 23/24, 95.8%; did not download the app: 21/24, 87.5%), suggesting that these factors were not barriers to completion.

**Conclusions:**

Despite self-reported intention to use the app, smartphone expertise, and sufficient facilitating conditions for smartphone use, implementation of the mobile app was infeasible for daily practice. This was due to a combination of technical problems, factors related to the implementation process, and the population of interest having cognitive disorders or a dependency on caregivers for mobile technology.

## Introduction

As a result of an aging population, the global incidence of hip fractures has been increasing with an estimated 6.25 million per year expected by 2050 [1,2]. In the Netherlands, 19,000 patients with hip fractures are treated annually [3,4]. To improve the quality of care for elderly patients with fractures of the hip, the multidisciplinary Centre for Geriatric Traumatology was established in 2008 at the Department of Trauma Surgery at Ziekenhuisgroep Twente hospital (located in Almelo and Hengelo in the Netherlands). Approximately 300 hip fracture patients are treated annually in this center [4]. To improve the quality of care among patients with fractures of the hip nationwide, the Dutch Hip Fracture Audit was established in 2016. The Dutch Hip Fracture Audit [5] monitors quality of care using indicators for quality of hospital stay, 3-month functional outcome, and 1-year mortality. Some of these quality indicators have been formulated by the Health and Youth Care Inspectorate and are mandatory; living situation of the patient, prefracture mobility score, and the Katz Index of Independence in Activities of Daily Living score are currently gathered during scheduled 3-month follow-up visits to the outpatient clinic.

The proportion of patients who register to provide information regarding functional recovery is poor; only 30.7% of Dutch Hip Fracture Audit registrations are completed [5]. Due to age or health-related factors, patients do not visit the outpatient clinic for their scheduled 3-month follow-up. Poor registration may result in a suboptimal monitoring of quality of care. In contrast, the 3-month registration was completed by 89.0% of the patients in the Centre for Geriatric Traumatology. This higher percentage was achieved by using an active telephone approach for patients who missed or canceled their outpatient appointments; however, the active approach was time consuming and inefficient. Mobile apps may offer an opportunity for improvement. Mobile app use to remotely monitor patients who have a low risk of postoperative complications has been investigated in multiple studies [6-10] which have concluded that mobile apps were useful for following up with patients who had a low risk of postoperative complication and with patients from 18 to 82 years of age who had undergone day-procedures. To our knowledge, no studies have investigated the use of mobile apps for the follow-up of patients with fractures of the hip.

There has been ongoing worldwide interest in home telemonitoring to support the health and vitality of the community-dwelling elderly population which has led to promising strategies for improving health care and health management [11-13]. Despite interest in the use of home telemonitoring, the literature mostly consists of pilot or feasibility studies. Real-world use and acceptance of home telemonitoring in daily care in older patient populations have mainly been studied in patients with chronic heart failure and have shown high acceptance of the technology using a 12-month survey [14,15]. In order to further optimize mobile app use among the elderly, a supportive theoretical framework has been recommended for iterative design of app implementation and evaluation [16]. These recommendations encompass multidisciplinary approaches, focus on end-user ease of use, and suggest starting with usability and feasibility testing in simulation environments [16-18]. In addition, during implementation, variation in levels of interest and technological literacy should be taken into consideration, especially among older adults [16].

The primary goal of this study was to investigate the real-world use of a mobile app for monitoring postoperative functional recovery after hip fracture. The secondary goals were to analyze mobile app usability and acceptance among elderly patients and their caregivers. Usability and acceptance were considered to facilitate conditions for use, but were not presumed to lead automatically to use.

## Methods

### App Development and Implementation

The mobile app platform was developed by technical experts, is currently used, and has previously been used in studies of postoperative outcome with a high rate of use [1]. A multidisciplinary team of health care professionals and technical experts developed a proof-of-concept version of the app that included specific adjustments for an older population of patients. A digital questionnaire consisting of indicators of quality of care from the Dutch Hip Fracture Audit was developed to remotely monitor postoperative functional outcome at 3 months. This questionnaire was implemented in the mobile app, and the technology was pretested with 2 patients with fractures of the hip who had been chosen at random.

### Participant Recruitment

**Figure 1:**
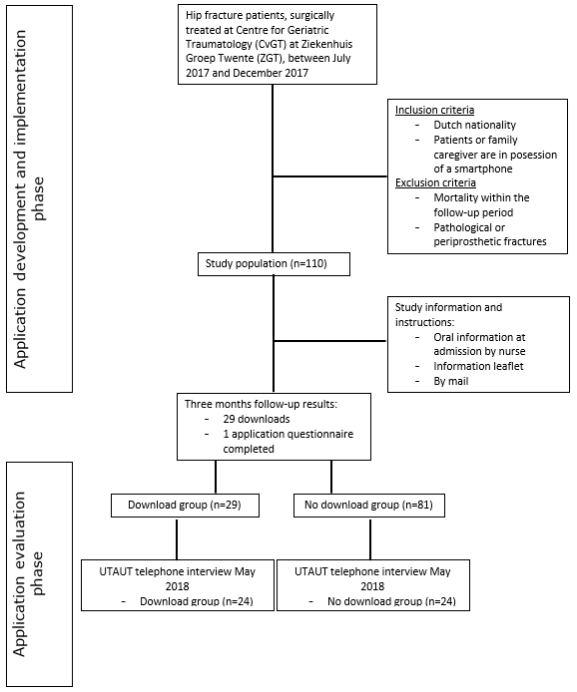
Study design flowchart.

Patients with a hip fracture who had undergone surgical treatment between July 2017 and December 2017 at the Centre for Geriatric Traumatology of the Department of Trauma Surgery at Ziekenhuisgroep Twente hospital were recruited to participate in the study and asked to download the app in addition to their regular 3-month outpatient visit (the recruitment process is summarized in Figure 1). The population of interest consisted of older adults, among whom information and communication technology literacy or low motivation to use technology may be factors that hinder implementation of a mobile app and which could suggest the need to focus on patient spouses in addition to the patients themselves [19]. For the purpose of this study, both patients and spouses who decided to participate were considered participants. During admission to the surgical ward of the Centre for Geriatric Traumatology, a nurse informed potential participants about the study, use of the app, and how to download instructions for using the app. After verbally providing informed consent, participants received an information leaflet and provided their email address for further information.

One week later, participants received a code by mail to activate the questionnaire in the downloaded app. Completion of the questionnaire was restricted to a period between 12 weeks and 18 weeks after their operation. A push notification with a request to complete the questionnaire was sent to the participant 12 weeks after they had been discharged from the hospital. A push notification was also sent to the health care provider at 17 weeks for unfilled questionnaires.

Completed questionnaires were saved in OpenLine (a specialized health care hosting center) in accordance with Dutch legislation with respect to security standards. The local researcher applied for the data from the hosting center. Participants were anonymized and coded using a study number without any reference to patient number or date of birth. Only the local researcher had access to the participant study numbers. All data were treated confidentially and saved to the secured hospital network with a password.

### Usability and Acceptance Questionnaire

To investigate usability and participant acceptance of the mobile app, an interview questionnaire was developed (Multimedia Appendix 1) based upon the Unified Theory of Acceptance and Use of Technology [20]; the model investigates user intentions and usage behavior in technology systems [20].

Two questions regarding participant recollection of the intended purpose of the study and feedback on the use of the app were added to the interview. These questions were added because we were interested in obtaining feedback on the app and on the duration of the interval from when the information was given (from July 2017 to December 2017) to when the telephone interview took place (in May 2018). A single researcher conducted all interviews. Participants were given the option to stop the telephone interview at any time.

### Data Collection

Data were collected from the clinical charts of the patients who participated themselves or whose caregivers participated. Age, gender, type of fracture, American Society of Anesthesiologists physical classification status, Charlson Comorbidity Index [21], dementia, prefracture Katz Index of Independence in Activities of Daily Living score [22], prefracture mobility score, and prefracture living situation were recorded as baseline characteristics. In April 2018, the app usage data from the hosting center were collected. Participants were divided into two groups—those who downloaded the app (use group) and those who did not download the app (nonuse group). Mobile app usability and acceptance telephone interviews were conducted with participants who could be reached by telephone within 3 attempts. The number of participants in both groups was adjusted to the lowest number of participants accessible by telephone of either group (use group, n=24); therefore, in the nonuse group, 24 participants were selected randomly. Participant answers were fully transcribed in individual and anonymized Office Word (version 2007; Microsoft Inc) documents and saved on a secure hospital server.

### Data Analysis

Statistical analyses were performed using SPSS software (version 22.0; IBM Corp). We used thematic analysis with a deductive theoretical approach to analyze the written answers to the recalled purpose of the study and feedback questions [23]. Identification of patterns and themes within the data was performed by one researcher, and a second researcher was consulted to reach agreement; the data were then coded by themes. Categorical data were analyzed using the chi-square test or Fisher exact test when appropriate (ie, Fisher exact test was used when frequency was less than 5). Functional outcomes were analyzed using two-tailed paired *t* tests. Continuous data were analyzed using two-tailed independent *t* tests. If significant differences were found in categorical variables with two or more subgroups, Pearson chi-square test was performed post hoc. *P*<.05 was considered statistically significant.

### Ethics

This study was been designated as an observational study not subject to the Dutch Medical Research Involving Human Subjects Act by the appropriate medical research ethics committees.

## Results

### Patient Characteristics

Categorical variables are described as numbers with corresponding percentages. Continuous variables are described as the mean with standard deviation, or for nonparametric data, as the median with interquartile range.

Patient characteristics are shown in Table 1. Patients with fractures of the hip (N=110) were a mean age of 80.5 (SD 10.4) years and were 71.8% (79/110) female and 28.2% (31/110) male. No significant differences were found between those who downloaded the app and those who did not download the app for age (*P*=.21), gender (*P*>.999), type of fracture (*P*>.999), American Society of Anesthesiologists physical classification status (*P*>.999), Charlson Comorbidity Index (*P*>.999), dementia (*P*=.05), prefracture Katz Index of Independence in Activities of Daily Living score (*P*=.10), prefracture mobility score (*P*=.10), and prefracture living situation (*P*=.73).

**Table 1:** Baseline patient characteristics.

Characteristics		All (N=110)		Downloaded app (n=24)		Did not download app (n=24)		Chi-square (*df*) or *t* test (*df*)		*P* value	
Age (years), mean (SD)		80.5 (10.4)		82.0 (8.7)		78.4 (10.8)		1.28 (46)		.21	
**Gender, n (%)**								1.0 (1)		>.999	
	Male		31 (28.2)		7 (29.2)		7 (29.2)				
	Female		79 (71.8)		17 (70.8)		17 (70.8)				
**Type of fracture, n (%)**								0.595 (2)		>.999	
	Neck of femur		64 (58.2)		13 (54.2)		14 (58.3)				
	Pertrochanteric		40 (36.4)		10(41.7)		10 (41.7)				
	Subtrochanteric		6 (5.5)		1 (4.2)		0 (0.0)				
**ASA^a^ physical status classification, n (%)**								1.0 (1)		>.999	
	1-2		40 (36.4)		9 (37.5)		9 (37.5)				
	3-4		70 (63.6)		15 (62.5)		15 (62.5)				
**Charlson Comorbidity Index, n (%)**								1.0 (3)		>.999	
	0-1		32 (29.1)		7 (29.2)		8 (33.3)				
	2-3		13 (11.8)		2 (8.3)		3 (12.5)				
	>4		6 (5.4)		1 (4.2)		0 (0.0)				
	Unknown		59 (53.6)		14 (58.3)		13 (54.2)				
Dementia, n (%)		13 (11.8)		0 (0.0)		5 (20.8)		0.06 (1)		.05	
Prefracture Katz ADL^b^ score (out of 6), median (IQR)		1.0 (2.0)		1.2 (1.6)		2.2 (2.3)		—		.10	
**Prefracture mobility score, n (%)**								0.578 (4)		.73	
	Freely mobile without aids		40 (36.4)		8 (33.3)		6 (25.0)				
	Mobile outdoors with one aid		2 (1.8)		1 (4.2)		0 (0.0)				
	Mobile outdoors with two aids or frame		30(27.3)		8 (33.3)		7 (29.2)				
	Some indoor mobility but never goes outside without help		36 (32.7)		7 (29.2)		10 (41.7)				
	No functional mobility (using lower limbs)		1 (0.9)		0 (0.0)		1 (4.2)				
	Unknown		1 (0.9)		0 (0.0)		0 (0.0)				
**Prefracture living situation, n (%)**								0.327 (2)		.50	
	Independent		87 (79.1)		21 (87.5)		19 (79.2)				
	Care home		7 (6.4)		2 (8.3)		1 (4.2)				
	Nursing home		14 (12.7)		1 (4.2)		4 (16.7)				
	Protected housing		2 (1.8)		0 (0.0)		0 (0.0)				

^a^ASA: American Society of Anesthesiologists.

^b^Katz ADL: Katz Index of Independence in Activities of Daily Living.

### App Use

Of the participants (29/110, 26.4%) who downloaded the mobile app, only 1 (1/29, 3.4%) completed the app questionnaire.

### Interviewed Participants

#### Characteristics

Participants characteristics of those who participated in the telephone interviews are presented in Table 2. In the use group (the subset of the group who downloaded the app), 95.8% (23/24) self-reported as expert level, and 87.5% (21/24) participants in the nonuse group (the subset of the group who did not download the app) self-reported as expert level. The groups showed significantly differences for smartphone usage of 5 to 10 years (use: 0/24, 0.0%; nonuse: 8/24, 33.3%; *P*=.004) and more than 10 years (use: 22/24, 91.7%; nonuse: 15/24, 62.5%; *P*=.02).

**Table 2:** Comparison of baseline characteristics between the use (participants downloaded the app) and nonuse (participants did not download the app) groups.

Variables		Both groups (n=48)	Use (n=24)	Nonuse (n=24)	Chi-square (*df*) or *t* test (*df*)	*P* value
Age (in years), mean (SD)		57.3 (10.3)	56.9 (9.8)	57.8 (10.9)	–0.279 (46)	.78
**Gender, n (%)**					1.0 (1)	>.999
	Male	14 (29.2)	7 (29.2)	7 (29.2)		
	Female	34 (70.8)	17 (70.8)	17 (70.8)		
**Relation to patient, n (%)**					0.133 (4)	.14
	Patient self	5 (10.4)	3 (12.5)	2 (8.3)		
	Partner	5 (10.4)	1 (4.2)	4 (16.7)		
	First-degree relative	34 (70.8)	20 (83.3)	14 (58.3)		
	Second-degree relative	3 (6.3)	0 (0.0)	3 (12.5)		
	Other	1 (2.1)	0 (0.0)	1(4.2)		
**Smartphone experience (years), n (%)**					0.008 (2)	.004
	<5	3 (6.3)	2 (8.3)	1 (4.2)	0.551 (1)	>.999
	5-10	8 (16.7)	0 (0.0)	8 (33.3)	0.002 (1)	.004
	>10	37 (77.1)	22 (91.7)	15 (62.5)	0.016 (1)	.04
Use of apps on a smartphone, n (%)		48 (100)	24 (100)	24 (100)	—	>.999
Self-registered expert level, n (%)		44 (91.7)	23 (95.8)	21 (87.5)	0.296(1)	.61

#### Questionnaire Results

Questionnaire results are presented in Multimedia Appendix 2. Among the use group, 95.8% (23/24) of participants had the intention of completing the app questionnaire; 41.7% (10/24) of the nonuse group had the intention of downloading the mobile app. In the nonuse group, 54.2% (13/24) stated that they were not informed during admission at the hospital or by mail of the app; 4% (1/24) had no intention of downloading the app. Therefore, no difference in expectancy determinants were calculated between the groups, and no answers were considered as blank.

#### Thematic Analysis

A thematic analysis was conducted to evaluate patient recollection of the study’s purpose. Participant responses (transcribed excerpts are presented in Multimedia Appendix 3) resulted in five themes: functional monitoring, replacement of the outpatient appointment, evaluation of participant satisfaction, no idea or not sure, and other. Correct answers for patient recollection of the study’s purpose were defined as those classified within the themes of functional monitoring and future replacement of the outpatient appointment.

The study purpose was correctly remembered by 62.5% (15/24) of the use group participants compared to only 20.8% (5/24) in the nonuse group; 50% (12/24) of the participants in the use group said that they did not receive a smart phone notification with the request to complete the questionnaire which suggested a suboptimal implementation process.

## Discussion

### Principal Findings

Completion of 3-month mandatory functional monitoring is poor among patients with fractures of the hip, which may result in a suboptimal monitoring of quality of care. This single-center pilot study to investigate the use and to analyze the usability and acceptance of a mobile app for monitoring postoperative functional recovery after hip fracture revealed poor results for actual use of the mobile app despite high self-reported intention to use the mobile app, high self-reported expertise in using mobile apps, and conditions that facilitated the use of mobile apps. This suggests that participants had the goal of using the mobile app, but that better support was needed to properly implement the technology in health care.

For many years, apps have been regarded as an alternative to paper questionnaires, but the use of apps may have difficulties as well, especially when implemented in a population of community-dwelling older patients [16]. This study demonstrated implementation difficulties; only 26.4% (29/110) participants downloaded the mobile app. This demonstrated that implementation of the app may have required that sufficient attention be given to education of the community-dwelling older patient users.

The low percentage of app downloads could partially be explained by an inability of the patients or caregivers to correctly remember the information that was provided to them in the hospital possibly as a result of stress [24]. Receiving information in a state of stress has been associated with suboptimal information processing and reduced cognitive efficiency [25,26]; therefore, correct timing of information provision is essential. This study provided both oral and written information, but more emphasis should be given to written information or video instructions, as this has been shown to lead to better information retention [27]. Among participants who are elderly, an inverse correlation has been reported between age and recall of medical information which could also have influenced the findings of this study [24,28]. The 3-month time period between when the information was provided and when the questionnaire was to be completed which also could have negatively affected information recall and recollection of the study’s purpose.

One participant completed the app questionnaire after downloading the app. This participant showed an active approach by contacting the app developers and completed the questionnaire with assistance from the developers.

A high percentage of the participants (34/48, 70.8%) who were interviewed were caregivers who were first-degree relatives of the patient. Study information was provided independently of whether a caregiver was present at the time of information provision; therefore, it is possible that some first-degree relatives were not provided with the study information if they were absent during recruitment.

The telephone interview findings demonstrated that many in the use group had the intention of completing the questionnaire. This indicates that those participants were motivated to complete the app questionnaire. In the nonuse group (11/24, 45.8%), participants remembered the study, and 10 out of the 11 intended to download the app. Given this result, there seems to be a good level of intention in both groups. Facilitating conditions, such as facilitated help, were high in both groups and were not a restrictive factor for app usage [29]. Some participants in the nonuse group (13/24, 54.2%) were unable to remember the study, and they could not complete the interview. Difficulties in patients or caregiver recollection of study information may have been influenced by the previously noted patient-related factors such as cognitive impairment, anxiety, or stress [24]. Approaching multiple caregivers when providing information and conducting the telephone interview may also be a reason for some participants reporting that they did not remember the study. Respondents (18/48, 41.6%) also reported technical problems. The app developers suggested the start-up phase of the app as a possible explanation for the technical problems. The developers also suggested that a lack of received notifications could have been as a result of participants not enabling the appropriate permissions for notifications when downloading the app. Providing help in the hospital with downloading of the app could assist with this issue. Another way to decrease the frequency of technical problems while also optimizing usability and acceptance would be to frequently evaluate the mobile app during the implementation process [16].

### Recommendations

Findings revealed intention to use the mobile app, but very low actual usage. The use of a mobile app as it was implemented in this study was not feasible, but the study findings suggested a potential for use if implemented properly. First, technical issues should be solved, and a helpdesk should be made available. Second, it is recommended to involve participants in the development and implementation phases—doing so can optimize ease of use and acquiring feedback during implementation is a feasible goal. Third, information provision needs to be optimized in terms of timing and method of dissemination. It is important to supply additional information after discharge in order to prevent low download rates as a result of patient or caregiver stress during admission [27]. Written information, video instructions, or fact sheets are preferred to oral information [2,3]. Fourth, in studies involving caregivers, a single contact person is recommended.

### Limitations

Selection bias in the downloading group represents a threat to validity, as patients or caregivers already intended to participate in the study by downloading the app.

### Conclusions

The use of a mobile app to monitor 3-month postoperative functional outcome of hip fracture was low. Despite intention, expertise, and sufficient facilitating conditions for using smartphones, the implementation of the mobile app in this study was demonstrated to be infeasible. Reasons for this included a technical problem, the implementation process, and population of interest having cognitive disorders or a dependency on caregivers for mobile technology.

## Appendix

Multimedia Appendix 1 Telephone interview questionnaire.

Multimedia Appendix 2 Questionnaire results.

Multimedia Appendix 3 Excerpts from participant responses.
