# Supplementary material for: Mobile App for Monitoring 3-Month Postoperative Functional Outcome After Hip Fracture: Usability Study
Source: JMIR Hum Factors. 2020 Sep 14;7(3):e16989. doi: 10.2196/16989 (PMC7522745; doi:10.2196/16989)
Supplement: Multimedia Appendix 1 [file humanfactors_v7i3e16989_app1.docx]

## Appendix A. Telephone interview

Age:

Gender:

Relation to patient:

**Study purpose**

*Could you explain to me why you were asked to complete the application at the three-month follow-up after a hip fracture?*

**Behavioural Intentions**

Not download group: *Did you intend to download the application?*

Download group*:* *Did you intend to complete the questionnaire?*

**Participant feedback**

No download group: *What is the reason you did not download the application?*

Download group: *What is the reason you did not complete the questionnaire?*

**Voluntariness of Use**

*You felt obliged to participate in the study?*

**Experience**

*Do you use a mobile phone?*

*For how long have you been using a mobile phone?*

*Which functions of a mobile phone do you use?*

*Do you will find yourself experienced in the use of a mobile phone?*

**Performance Expectancy**

*Did you expect that the mobile application would be useful in the follow-up of a hip fracture?*

*As explained on the surgical ward: for several years we have been monitoring hip fracture patients after three months. Unfortunately, we registered poor result in outpatient visits after three months. There are, of course, several reasons such as difficulties for patients to come at the outpatient clinic or they wellbeing is not good enough to visit the outpatient clinic. To ensure we could still monitor these ‘loss to follow-up’ patients, we introduced this application. Were you expecting that using a mobile application would help to monitor our patients?*

**Effort Expectancy**

*Did you expect the mobile application was easy to use?*

**Social Influence**

*Did you talked about the application with relatives?*

*Have they encouraged you to use the application?*

*Did you expect people like family or friends would be helpful in using the mobile application?*

**Facilitating Conditions**

*Could you ask for help when using the application?*

*Have you asked for help when using the application?*
