# Supplementary material for: Mobile App for Monitoring 3-Month Postoperative Functional Outcome After Hip Fracture: Usability Study
Source: JMIR Hum Factors. 2020 Sep 14;7(3):e16989. doi: 10.2196/16989 (PMC7522745; doi:10.2196/16989)
Supplement: Multimedia Appendix 2 [file humanfactors_v7i3e16989_app2.docx]

**Appendix B.** Questionnaire results

|  | Total  (n=48) | Download group (n=24 ) | No download  group (n=24) | p-value |
| --- | --- | --- | --- | --- |
| Intention to respond the application questionnaire; |  |  |  |  |
| Yes; n (%) | 33 (68.8) | 23 (95.8) | 10 (41.7) |  |
| No intention; n (%) | 1 (2.1) | 0 (0.0) | 1 (4.2) |  |
| Blanco; n (%) | 14 (29.2) | 1 (4.2) | 13 (54.2) |  |
| Voluntariness of use |  |  |  |  |
| Yes; n (%) | 34 (70.8) | 23 (95.8) | 11 (45.8) |  |
| Blanco; n (%) | 14 (29.2) | 1 (4.2) | 13 (54.2) |  |
| Performance Expectancy |  |  |  |  |
| Expected useful in follow-up |  |  |  |  |
| Yes; n (%) | 31 (64.6) | 22 (91.7) | 9 (37.5) |  |
| No; n (%) | 2 (4.2) | 1 (4.2) | 1 (4.2) |  |
| Blanco; n (%) | 15 (62.5) | 1 (4.2) | 14 (58.3) |  |
| Expected improved control of participants |  |  |  |  |
| Yes; n (%) | 32 (66.7) | 22 (91.7) | 10 (41.7) |  |
| No; n (%) | 1 (2.1) | 1 (4.2) | 0 (0.0) |  |
| Blanco; n (%) | 15 (62.5) | 1 (4.2) | 14 (58.3) |  |
| Effort Expectancy |  |  |  |  |
| Yes; n (%) | 31 (64.6) | 22 (91.7) | 9 (37.5) |  |
| No; n (%) | 1 (2.1) | 1 (4.2) | 0 (0.0) |  |
| Blanco; n (%) | 16 (33.3) | 1 (4.2) | 15 (62.5) |  |
| Social Influence |  |  |  |  |
| Talked about application |  |  |  |  |
| Yes; n (%) | 9 (18.8) | 7 (29.2) | 2 (8.3) |  |
| No; n (%) | 26 (54.2) | 17 (70.8) | 9 (37.5) |  |
| Blanco; n (%) | 13 (27.1) | 0 (0.0) | 13 (54.2) |  |
| Encouraged |  |  |  |  |
| Yes; n (%) | 9 (18.8) | 7 (29.2) | 2 (8.3) |  |
| Blanco; n (%) | 39 (81.3) | 17 (70.8) | 22 (91.7) |  |
| Expected help |  |  |  |  |
| Yes; n (%) | 30 (62.5) | 21 (87.5) | 9 (37.5) |  |
| No; n (%) | 1 (2.1) | 1 (4.2) | 0 (0.0) |  |
| Blanco; n (%) | 17 (35.4) | 2 (8.3) | 15 (62.5) |  |
| Facilitating Conditions |  |  |  |  |
| Facilitated help |  |  |  |  |
| Yes; n (%) | 45 (93.8) | 22 (91.7) | 23 (95.8) | 1.000 |
| No; n (%) | 3 (6.3) | 2 (8.3) | 1 (4.2) |  |
| Used help (n=24) |  |  |  |  |
| Yes; n (%) |  | 3 (12.5) |  |  |
| No; n (%) |  | 21 (87.5) |  |  |
| Participant reminded study purpose; n (%) |  |  |  |  |
| Monitoring functioning | 12 (25.0) | 7 (29.2) | 5 (20.8) |  |
| Replacement of an outpatient appointment | 8 (16.7) | 8 (33.3) | 0 (0.0) |  |
| Evaluation patients' satisfaction | 5 (10.4) | 3 (12.5) | 2 (8.3) |  |
| No idea / not sure | 21 (43.8) | 6 (25.0) | 15 (62.5) |  |
| Other (telephone interview or information) | 2 (4.2) | 0 (0.0) | 2 (8.4)) |  |
| Participant feedback; n (%) |  |  |  | <0.001 |
| No reminder | 12 (25.0) | 12 (50.0) | 0 (0.0) | <0.001 |
| No questionnaire available | 2 (4.2) | 2 (8.3) | 0 (0.0) | 0.489 |
| Other technical problems | 6 (12.5) | 5 (20.8) | 1 (4.2) | 0.188 |
| Forgotten | 13 (27.1) | 5 (20.8) | 8 (33.3) | 0.330 |
| Unknown | 15 (31.3) | 0 (0.0) | 15 (62.5) | <0.001 |

*n, number of participants.*

*Differences in baseline characteristics between the download and the no download group were tested.*
