# Supplementary material for: Mobile App for Monitoring 3-Month Postoperative Functional Outcome After Hip Fracture: Usability Study
Source: JMIR Hum Factors. 2020 Sep 14;7(3):e16989. doi: 10.2196/16989 (PMC7522745; doi:10.2196/16989)
Supplement: Multimedia Appendix 3 [file humanfactors_v7i3e16989_app3.docx]

**Appendix C.** Reminded study purpose

Download group

*'So that you can easily follow and monitor patients. Beforehand we could fill in the app. If I go to the outpatient appointment with my mother, we will have an easier conversation because certain things were known through the app.’ (Participant 26)*

*‘I think it was a questionnaire. I do not know exactly anymore .. to fill in a questionnaire for three months. It was about my wife, what our opinion was about the treatment.’ (Participant 9)*

*‘Because she had a hip fracture… What I can still remember is that they wanted to see how the recovery was. And then we had to complete it after three months. ' (Participan79)*

*‘Yes, it would be easier because if you answered this, she would not have to come to the outpatient appointment anymore.’ (Participant 56)*

*‘Because there was a check up after three months. And if everything still was fine, time was spent unnecessarily on outpatient visits.MEER VERTALEN (Participant 43)*

*'It had to do with the fact that mommy did not have to go to the check up and instead we could complete an app. Yes, that it would prevent unnecessary travel. ' (Participant92)*

*To prevent patients to come to the hospital after certain months, which also could be done with a questionnaire. (Participant 70)*

*Was to see if everything went well and to see if there were unclear things. (P84)*

*'In the context of an evaluation study, I thought.' (Participant 76)*

No download group

*‘The app was about following the operation and that I could indicate whether something had changed. It has been so long ago that I cannot remember it well.’ (Participant 13)*

*‘That you can see from a distance how the recovery progressed.’ (Participant 83)*

*‘No idea’ (Participant 37)*

*‘I could see information there, or something else?’ (Participant 41)*

*"No, I cannot remember. It was a very busy time at Christmas at that time. I think that is why? If I had thought about it, I might have downloaded it.’ (Participant 106)*

*‘I can vaguely remember something. Do not know anything specific about it anymore.’ (Participant 22)*

*‘I remember something, but I do not know exactly what and how.’ (Pparticipant 51)*

*‘I can remember. If I remember correctly; about the evaluation and what we thought about it.’ (Participant 73)*

*‘No, no idea why.’ (Participant 38)*
